# Trust, ethical, and learning impact of artificial intelligence feedback in English education

**DOI:** 10.3389/fpsyg.2026.1809661

**Published:** 2026-04-16

**Authors:** Amani A. Binjwair, Ashah A. Alsaleh

**Affiliations:** 1 Department of Curriculum and Instruction, College of Education, Prince Sattam bin Abdulaziz University, Al-Kharj, Saudi Arabia; 2 Department of English Language, College of Arts, Jouf University, Jouf, Saudi Arabia

**Keywords:** AI feedback, trust in AI, ethical issues in AI, English education, learning outcomes, technology acceptance, student engagement, AI in education

## Abstract

**Background:**

Artificial intelligence-generated feedback (AI-GF) is increasingly used in computer-assisted English as a Foreign Language formative assessment, yet learner engagement with such feedback varies considerably. Previous research has focused largely on technical effectiveness, providing limited insight into how trust, ethical concerns, and learner engagement shape educational value. This gap restricts informed and responsible integration of artificial intelligence feedback in higher-education language learning.

**Objectives:**

This study investigates how trust, ethical concerns, clarity, and learner engagement influence the perceived usefulness and future readiness associated with AI-GF in English as a Foreign Language (EFL) learning.

**Methods:**

Drawing on technology acceptance and feedback literacy frameworks, a learner centered conceptual model was developed. Survey data were collected from 255 university-level EFL learners enrolled in higher education institutions. The data was analyzed using Partial Least Squares Structural Equation Modelling (PLS-SEM) to examine relationships among trust, ethics, engagement, clarity, perceived usefulness, and future readiness. Learners’ prior experience with artificial intelligence (AI) and academic maturity were included to capture variation in engagement patterns.

**Results and conclusion:**

The findings indicate that trust is the strongest predictor of learner engagement with AI-GF, while ethical concerns significantly undermine trust. Engagement operates as the principal mechanism linking AI-GF to perceived usefulness and readiness for future learning, whereas clarity alone is necessary but insufficient to support meaningful learning outcomes. These results extend existing models by positioning engagement as a central outcome and integrating trust and ethics as foundational determinants. Pedagogically, the study highlights the importance of ethical transparency, trust-building practices, and structured learner guidance in maximizing the educational impact of AI-GF in computer-assisted language learning contexts.

## 1 Introduction

The AI tools in higher education have transformed formative assessment, particularly in the EFL context. Generative AI systems such as ChatGPT, alongside automated writing evaluation platforms, offer immediate, individualized, and scalable feedback, extending instructional support beyond traditional classroom limitations (Alshamy et al., 2025; Shahzad et al., 2024). Despite these potential benefits, learners’ experiences with AI-GF vary considerably and are influenced by factors such as the feedback source (human versus AI), design features (clarity, specificity, and immediacy), learner trust, engagement, and sociocultural context (Chan and Hu, 2023; Zhang et al., 2024).

Empirical research has demonstrated that AI-GF can enhance revision frequency, writing accuracy, and learner autonomy (Mekheimer, 2025; Muthmainnah et al., 2024). However, EFL students often perceive instructor feedback as more effective in promoting higher-order skills, personalized guidance, and authentic interactions (Aljasser, 2025). Attributes such as specificity and clarity critically influence students’ perceived usefulness of feedback, with actionable, targeted comments being preferred over generic suggestions (Aljasser, 2025). Furthermore, ethical, cultural, and contextual factors, including fairness, bias, over-reliance, and alignment with local pedagogical practices, can moderate AI-GF’s effectiveness (Alshamy et al., 2025; Aljabr and Al-Ahdal, 2024).

Despite AI-GF’s growing adoption, previous studies reveal several critical gaps. Research has predominantly focused on secondary school learners or the general ESL population, leaving higher-education EFL students underexplored (Hillmayr et al., 2020; Li et al., 2024; Moussa et al., 2024). Moreover, many studies have examined isolated components of AI feedback, such as grammar correction and revision frequency, rather than its holistic impact on learning outcomes, engagement, and self-regulated learning (Luo and Yusuf, 2025; Mekheimer, 2025; Muthmainnah et al., 2024). Furthermore, ethical, cultural, and integration issues are often treated superficially, limiting our understanding of how AI-GF interacts with pedagogical, institutional, and learner-specific factors (Alghamdy, 2023; Aljabr and Al-Ahdal, 2024; Alshamy et al., 2025; Granström and Oppi, 2025). Additionally, evidence of the interplay between AI feedback characteristics such as clarity, specificity, and immediacy and students’ perceptions, engagement behaviors, and academic outcomes in higher-education EFL contexts remains limited (Huang and Mizumoto, 2025).

Addressing these gaps requires an integrated perspective that links learners’ perceptions, engagements, and ethical considerations with concrete learning outcomes. Drawing on Feedback Literacy Theory (Carless and Boud, 2018), the Technology Acceptance Model (TAM) (Davis, 1989), and the principles of ethical AI in education (Aljabr and Al-Ahdal, 2024; Holmes et al., 2022), this study conceptualizes AI-GF as a mediation tool. Feedback characteristics such as clarity, specificity, and immediacy influence learners’ trust, perceived usefulness, and engagement, which in turn shape outcomes such as revision behavior, writing quality, and self-regulated learning. Ethical and contextual factors, including fairness, bias, privacy, and cultural relevance, moderate these relationships, while AI literacy and institutional support serve as enabling conditions for effective adoption (Corbin et al., 2025a; Luo and Yusuf, 2025; Venter et al., 2025). Accordingly, this study investigated the following research questions:

1. What are higher-education EFL students’ perceptions of AI-GF’s clarity?
2. To what extent do students trust the accuracy and objectivity of the AI-GF?
3. How useful is AI-GF for enhancing student learning and academic performance?
4. How do students engage with AI-GF in formative assessment?
5. What ethical concerns do students have regarding the use of AI-GF?
6. Do student perceptions of AI-GF differ according to their level of AI tool use?
7. Does the year of study significantly affect student engagement with AI-GF?

Guided by these research questions, this study proposes a set of theory-driven hypotheses specifying the structural relationships among perceived clarity, trust, engagement, perceived usefulness, ethical concerns, and future readiness. By addressing these questions, this study seeks to provide a comprehensive, evidence-based understanding of AI-GF in higher-education EFL contexts by informing pedagogical design, ethical practice, and the integration of AI tools in formative assessment.

## 2 Literature review

Integrating artificial intelligence (AI) into higher education has transformed formative assessment practices, particularly in the EFL context. AI-GF supports learners by providing timely and personalized comments on writing and other academic tasks (Huang and Mizumoto, 2025; Luo and Yusuf, 2025). Recent studies have further highlighted the expanding role of AI-driven technologies in shaping collaborative language learning environments and digital language pedagogy (John, 2025; John, 2026a). Prior research has highlighted benefits such as enhanced revision quality, increased learner motivation, and improved writing performance (Mekheimer, 2025; Pariyanto and Tungka, 2025). Despite these promising outcomes, existing studies have revealed critical gaps in understanding student perceptions, engagement, and ethical concerns, which are central to effective AI adoption in authentic learning environments.

### 2.1 Student perceptions and trust in AI feedback

Clarity and trust are foundational for feedback to be useful (Hattie and Timperley, 2007). Although studies have indicated that AI-generated comments are often clear and actionable, learners’ trust in their accuracy, objectivity, and pedagogical validity remains underexplored (Henderson et al., 2025). Students may question AI reliability, particularly when automated feedback conflicts with human evaluations (BinJwair and Alamer, 2025; Praveen et al., 2025). Most prior research has focused on performance outcomes or controlled writing tasks (Er et al., 2025; Tensen et al., 2025), often neglecting holistic perceptions of AI feedback in naturalistic classroom settings. Recent studies suggest that learners’ acceptance and engagement with AI-GF are influenced not only by perceived clarity but also by their prior experience with digital tools, trust in AI systems, and attitudes toward generative AI technologies (BinJwair, 2025; Jwair, 2025). Understanding these factors is critical for leveraging AI feedback effectively in higher education.

### 2.2 Perceived usefulness and learning impact

Perceived usefulness, a central construct within the Technology Acceptance Model (Davis, 1989; Naidoo, 2023), shapes whether learners act on feedback. Existing research has demonstrated AI’s potential to enhance writing quality, self-regulated learning, and task efficiency (Mirzaei and Khayer, 2025; Muthmainnah et al., 2024). However, most studies have emphasized short-term gains or isolated assignments rather than sustained engagement across multiple tasks. Consequently, the relationship between perceived usefulness and actual learning outcomes, including revision behaviors and skill development, remains insufficiently addressed. Integrating Feedback Literacy Theory (Carless and Boud, 2018) provides a theoretical lens through which to examine how learners interpret, act on, and internalize AI feedback beyond mere acceptance.

### 2.3 Engagement with AI feedback

Learner engagement is critical for meaningful feedback uptake (Bognár et al., 2024; Wang and Wu, 2025). Although AI tools can provide adaptive and immediate feedback, empirical studies have revealed mixed evidence on whether students critically reflect on or merely accept AI comments (Luo and Yusuf, 2025). Few studies account for contextual moderators, such as the year of study or prior AI tool exposure, which may influence engagement patterns. Bridging these gaps requires investigating how engagement behaviors mediate the relationship between AI feedback characteristics and learning outcomes, particularly in EFL higher-education settings.

### 2.4 Ethical concerns and responsible use

Despite the technological advancements, ethical considerations are often overlooked in AI feedback research. Concerns regarding bias, accuracy, overreliance, and privacy are increasingly recognized (Aljabr and Al-Ahdal, 2024; Alghamdy, 2023; Corbin et al., 2025b). Recent research has also examined educators’ perceptions of the role of artificial intelligence in academic integrity and pedagogical practices, highlighting the importance of responsible AI integration in educational contexts (Praveen et al., 2025). Besides, students’ willingness to engage critically in AI feedback may be influenced by such concerns. However, empirical evidence remains limited. Incorporating Ethical AI principles (Holmes et al., 2022) addresses this gap by framing students’ critical engagement as contingent on perceived fairness, transparency, and institutional support.

### 2.5 AI literacy and contextual factors

Institutional support and AI literacy are pivotal to effectively integrating AI feedback (Granström and Oppi, 2025; Chen, 2025). A recent study has also highlighted the broader impact of digital technologies and artificial intelligence on language teaching and learning environments (John, 2026b). Training students to interpret AI-generated comments enhances trust calibration, engagement, and feedback uptake (Venter et al., 2025; Huang and Mizumoto, 2025). However, few studies have systematically evaluated how such enabling conditions moderate the relationship between AI feedback characteristics and learning outcomes, leaving a significant gap in the understanding of how to support learners in diverse higher educational contexts.

## 3 Conceptual framework and hypothesis development

This study was guided by an integrated conceptual framework based on the Technology Acceptance Model (TAM) (Davis, 1989), the Unified Theory of Acceptance and Use of Technology (UTAUT) (Venkatesh et al., 2003), Feedback Literacy Theory (Carless and Boud, 2018; Molloy and Boud, 2013), and the principles of ethical AI in education (Alghamdy, 2023; Aljabr and Al-Ahdal, 2024; Holmes et al., 2022). These complementary perspectives provide a robust foundation for examining EFL students’ perceptions, engagements, and ethical concerns regarding AI-GF in formative assessment contexts.

The UTAUT framework proposes that individuals’ adoption of technological systems is influenced by factors such as performance expectancy, effort expectancy, social influence, and facilitating conditions (Venkatesh et al., 2003). Building on this perspective, the present study extends technology acceptance research by examining additional factors particularly relevant to AI-supported learning environments, including trust in AI-GF, ethical concerns regarding AI use, and learner engagement with automated feedback.

In line with prior research, AI-GF is conceptualized as a formative learning resource whose pedagogical value depends not solely on automation, but on learners’ perceptions of clarity, trustworthiness, and usefulness, as well as on their capacity for critical engagement with feedback (Corbin et al., 2025a; Hattie and Timperley, 2007; Henderson et al., 2025). Given the self-reported nature of the data, the framework focused on perceived learning and academic impact rather than objectively measuring achievement.

### 3.1 Perceived clarity and perceived usefulness

Perceived clarity refers to the extent to which the AI-GF is understandable, well-structured, and easy to interpret. Within the TAM, clarity corresponds conceptually to perceived ease of use, which is a key antecedent of perceived usefulness (Davis, 1989; Naidoo, 2023). AI feedback design studies indicate that clear, specific, and linguistically accessible feedback facilitates comprehension and revision, particularly in EFL contexts (Aljasser, 2025; Zhang et al., 2024). Empirical studies have consistently shown that when learners perceive feedback as clear, they are more likely to recognize its value in improving their academic work (Granström and Oppi, 2025).

*H1*: Perceived clarity of AI-GF has a significant positive effect on perceived usefulness.

### 3.2 Trust and engagement with AI-generated feedback

From the feedback literacy perspective, trust is a prerequisite for meaningful engagement with feedback. Learners are more likely to reflect on, apply, and integrate feedback when they perceive it as credible and fair (Jin et al., 2025; Ruegg, 2017).

Recent empirical work has demonstrated that trust in AI systems positively predicts student engagement, including reflective revision practices and sustained interactions with AI-GF (Huang and Mizumoto, 2025; Li et al., 2024; Wang and Wu, 2025).

*H2*: Trust in AI-GF is positively associated with student engagement with the feedback.

### 3.3 Engagement and perceived usefulness

Engagement with AI-GF encompasses reflective thinking, comparisons with instructor feedback, and goal setting during revision. The feedback literacy theory emphasizes that learning benefits arise from active feedback use rather than passive receipt (Heil and Ifenthaler, 2025; Molloy and Boud, 2013).

Studies in AI-enhanced EFL writing contexts have shown that students who critically engage in AI feedback report higher levels of perceived learning support and usefulness (Luo and Yusuf, 2025; Mirzaei and Khayer, 2025; Moussa et al., 2024). Therefore, this study proposes the following hypothesis:

*H3*: Engagement with AI-GF has a significant positive effect on perceived usefulness.

### 3.4 Ethical concerns and trust in AI-generated feedback

Trust in the AI-GF reflects learners’ confidence in the accuracy, objectivity, and reliability of AI systems. Trust is a crucial factor in AI-mediated educational environments, where students must rely on automated judgments to inform learning decisions (Qin et al., 2020). However, ethical concerns, including data privacy, algorithmic bias, inaccuracies, and overreliance, may undermine students’ trust in AI feedback (Alghamdy, 2023; Aljabr and Al-Ahdal, 2024; Corbin et al., 2025b). Studies in the EFL and higher-education contexts suggest that unresolved ethical issues negatively affect learners’ willingness to rely on AI-generated guidance (Alshamy et al., 2025; Shahzad et al., 2024).

*H4*: Ethical concerns have a significant negative effect on students’ trust in AI-GF.

### 3.5 Perceived usefulness, engagement, and future readiness

Perceived usefulness is a central construct in TAM and has been shown to predict continued technology use and readiness for further training (Davis, 1989; Granström and Oppi, 2025). In educational contexts, students who perceive AI feedback as useful are more likely to feel prepared to engage in AI-enhanced learning environments (Khan, 2025; Ouyang et al., 2023).

**Figure 1:**
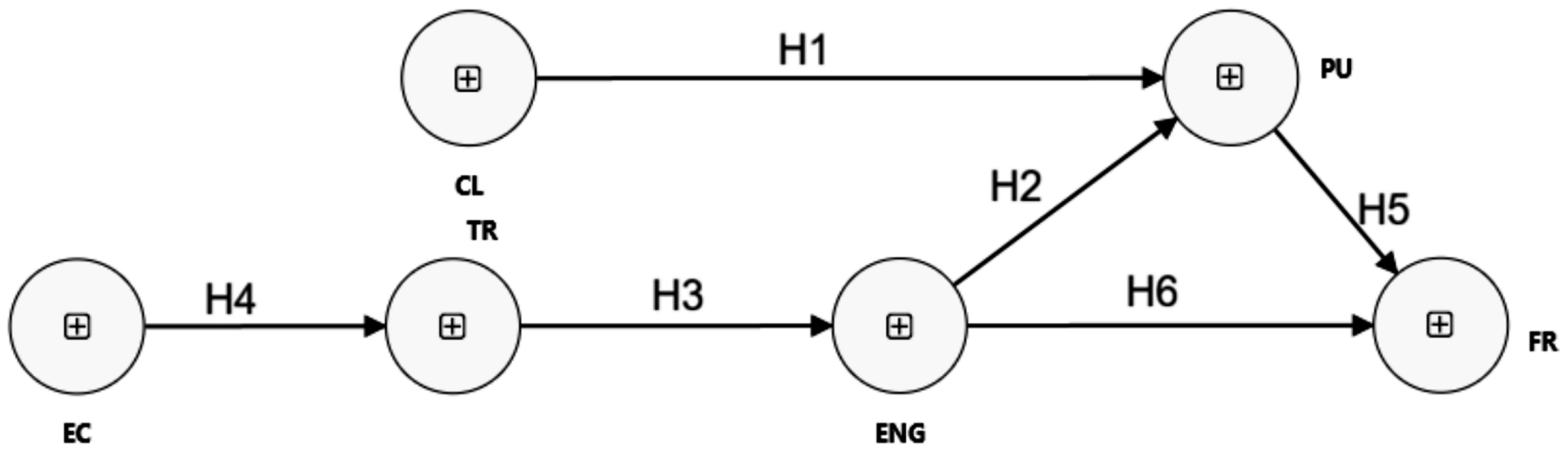
Conceptual framework diagram. The conceptual framework proposes how the characteristics of AI-GF, namely clarity, specificity, usefulness, and immediacy, influence students’ perceptions and trust in AI systems. Subsequently, these perceptions affect student engagement, revision behaviors, and learning outcomes. Ethical and contextual considerations, including bias, fairness, overreliance, and privacy, are modeled as moderating factors that shape the strength and direction of these relationships. Additionally, institutional AI-literacy initiatives and training are positioned as enabling conditions that support the responsible and effective integration of AI-GF in educational settings.

Beyond its usefulness, active engagement with AI feedback contributes to developing feedback literacy, self-regulation, and confidence, which are key components of future readiness (Bognár et al., 2024; Huang and Mizumoto, 2025) (See Figure 1).

*H5*: Perceived usefulness of AI-GF positively predicts students’ future readiness for AI feedback training.

*H6*: Engagement with AI-GF positively predicts students’ future readiness for AI feedback training.

### 3.6 Developmental and usage-based differences

Drawing on TAM extensions and engagement research, the framework also accounts for differences based on the AI tool usage level and year of study. Prior research indicates that familiarity with artificial intelligence tools influences perceived usefulness and trust (Li et al., 2024; Naidoo, 2023), whereas progression through higher education enhances feedback literacy and reflective engagement (Heil and Ifenthaler, 2025; Ruegg, 2017).

## 4 Methodology

### 4.1 Research design

This study employed a quantitative cross-sectional survey design to investigate EFL learners’ perceptions of trust, engagement, ethical concerns, and perceived learning impact regarding AI-GF in formative assessment. A survey design was chosen because it allows systematic and scalable measurement of latent constructs such as trust, perceived usefulness, engagement, and ethical considerations, consistent with prior research on AI in education (Chan and Hu, 2023).

This study employed convenience sampling due to practical constraints in accessing students across multiple courses. As a result, the sample was predominantly female (93.7%), which limits the generalizability of the findings. Therefore, future research should aim to recruit more balanced and diverse samples to explore whether these perceptions differ across gender and other demographic factors, thereby enhancing the robustness and applicability of the findings.

### 4.2 Participants

Participants were undergraduate EFL learners enrolled in a higher-education institution in Saudi Arabia. Eligibility required enrollment in an EFL course during the study period. Recruitment was conducted through the official university email. Ethical approval was obtained from the Institutional Review Board, participation was voluntary, informed consent was obtained electronically, and all responses were anonymized. The study posed minimal risk, relying solely on self-reported survey data.

The target population consisted of 632 students. A power analysis using G*Power 3.1.9.7, based on a medium effect size (f^2^ = 0.15), an alpha level of 0.05, and a statistical power of 0.80, indicated a minimum sample of 245 participants. To ensure robustness and account for potential outliers, 255 valid responses were collected and analyzed.

**Figure 2:**
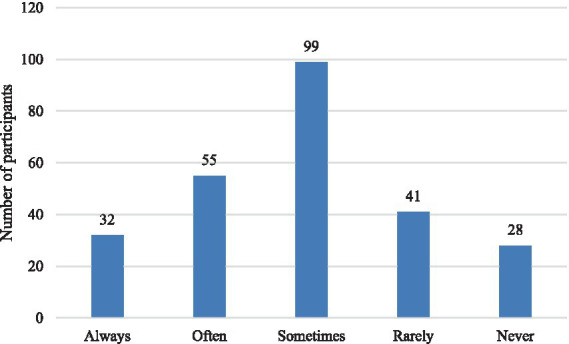
Distribution of the study sample by the use of AI in academic work.

**Figure 3:**
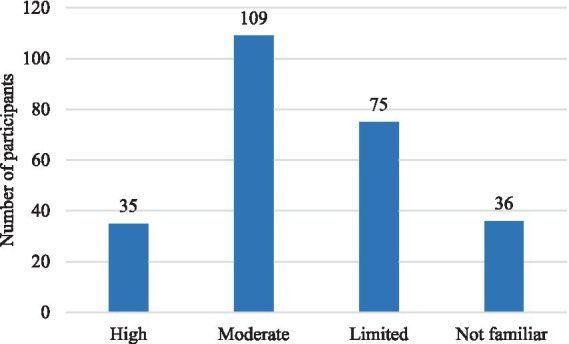
Distribution of the study sample by familiarity with AI for academic purposes.

Demographically, the majority of participants were female (93.7%), aged between 21 and 23 years (41.6%), and enrolled in bachelor’s programs (75.7%), with the largest subgroup in their third year of study (45.1%). Regarding prior experience with AI-GF, 31.4% of participants reported having received formal AI-GF within academic coursework. However, the remaining participants were not entirely unfamiliar with AI technologies. A substantial proportion of respondents reported moderate (42.7%) or high (13.7%) academic use of AI tools, indicating previous interaction with AI-based systems for tasks such as writing assistance, language learning, or other academic activities. This distinction between formal AI-GF experience and broader exposure to AI technologies provides transparency regarding participants’ backgrounds and ensures the validity of their responses concerning perceptions of AI-GF (See Table 1; Figures 2, 3).

**Table 1:** Distribution of the study sample by demographic characteristics.

Variable	Categories	*N*	%
Gender	Female	239	93.7
	Male	16	6.3
Age group	18–20	99	38.8
	21–23	106	41.6
	24–26	30	11.8
	27 and above	20	7.8
Academic program	Diploma	49	19.2
	Bachelor	193	75.7
	Postgraduate	13	5.1
Year of study	1st year	61	23.9
	2nd year	37	14.5
	3rd year	115	45.1
	4th year	42	16.5
Received AI-GF on academic work	Yes	81	31.4
	No	174	68.6

*N*, Sample size; %, Percentage distribution.

### 4.3 AI-generated feedback context

In this study, AI-GF refers to automated formative feedback produced by AI tools that analyze students’ written responses and provide suggestions for improvement. The feedback typically addressed grammatical accuracy, sentence clarity, vocabulary use, and overall coherence.

AI-GF was implemented as an additional formative assessment tool designed to support learners’ reflection on writing and guide improvements in language accuracy and clarity. By providing immediate, structured feedback, AI-GF aimed to complement traditional teacher feedback and foster learner autonomy.

### 4.4 Instrumentation and measurement of constructs

Data were collected using a structured questionnaire, grounded in prior research on AI-GF and formative assessment in higher education (Henderson et al., 2025; Xu et al., 2025). Data collection occurred from November 19 to December 13, 2025. The instrument was theory-driven, focusing on learner perceptions, engagement, and ethical considerations, rather than tool-specific evaluations or direct experience with AI-GF. In SEM studies, especially using PLS-SEM, perception-based measures are widely accepted for assessing latent constructs even when direct experience is limited (Davis, 1989; Venkatesh et al., 2003; Venkatesh and Davis, 2000).

The questionnaire comprised seven sections: demographic information, AI usage, perceptions and trust, comparative perspectives, learning impact, engagement with AI-GF, ethical concerns, and future readiness and training. All perceptual items were measured on a five-point Likert scale (1 = strongly disagree to 5 = strongly agree). The constructs included:

- Trust in AI feedback: Learners’ confidence in the reliability and pedagogical value of AI-GF.
- Ethical concerns: Learners’ perceptions of potential risks related to AI use, including fairness, bias, privacy, overreliance, and possible impact on critical thinking. Items were measured as a single construct.
- Engagement: Cognitive and behavioral interaction with AI-GF, including attention, reflection, and application of feedback.
- Perceived learning impact: Learners’ perceptions of AI-GF’s contribution to language learning improvement.
- Perceived clarity of AI-GF, perceived usefulness for learning, future readiness, and training were also measured.

Survey items were adapted from validated instruments in educational technology, feedback, and applied linguistics research (Alshamy et al., 2025; Henderson et al., 2025; Xu et al., 2025). Minor wording adjustments ensured contextual relevance for undergraduate EFL learners while maintaining fidelity to the original theoretical constructs. Appendix 1 summarizes the constructs, operational definitions, and measurement sources to enhance transparency and replicability.

### 4.5 Data collection procedure

Data collection was conducted online via structured surveys administered to eligible participants. Participation was voluntary, anonymous, and posed minimal risk. The survey was open for approximately four weeks, and reminders were sent to ensure adequate response rates. Responses were screened for completeness and validity, resulting in 255 usable responses for analysis.

### 4.6 Data analysis

Data analysis was conducted in two stages: descriptive analysis and structural equation modelling (SEM). Descriptive statistics (frequencies and percentages) were used to summarize participants’ demographic characteristics and patterns of AI tool usage. Visual representations (bar charts) were used to illustrate key distributions.

To test the hypothesized relationships and evaluate the proposed conceptual framework, partial least squares structural equation modelling (PLS-SEM) was performed using SmartPLS (version 4.1.0.3). PLS-SEM was selected because of its suitability for theory development and prediction-oriented research, robustness in handling complex models with multiple latent constructs, and appropriateness for applied linguistics and educational technology research (Alamer, 2025; Hair et al., 2019). The analysis followed a structured two-stage procedure, consistent with established PLS-SEM guidelines, involving evaluating the measurement model, followed by assessing the structural model (Alamer, 2025; Hair et al., 2019).

#### 4.6.1 Measurement model assessment

The reflective measurement model was evaluated following established PLS-SEM guidelines (Alamer, 2025; Hair et al., 2019). Internal consistency reliability was assessed using Cronbach’s alpha and composite reliability (CR), with values ≥ 0.70 indicating acceptable reliability. Convergent validity was examined through average variance extracted (AVE), with values ≥ 0.50 demonstrating adequate construct convergence.

Discriminant validity was assessed using the heterotrait-monotrait ratio of correlations (HTMT), with values below 0.85 indicating adequate discriminant validity. To assess potential multicollinearity among indicators, variance inflation factor (VIF) values were examined, with values below 5.0 indicating no critical collinearity concerns. These procedures align with the recent applications of SEM in second language research and construct validation studies (Alamer and Alrabai, 2024; Alamer et al., 2023).

#### 4.6.2 Structural model assessment

**Figure 4:**
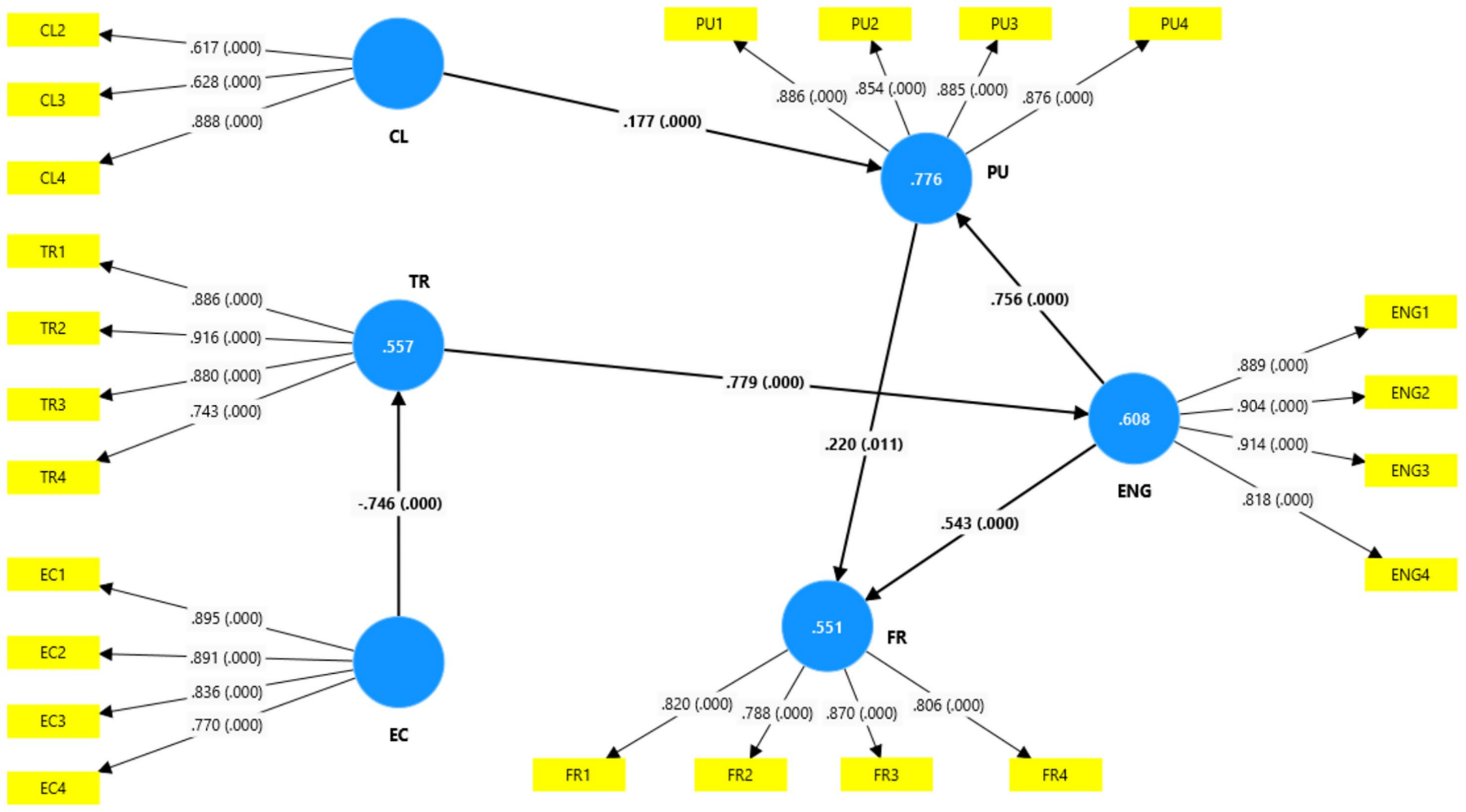
The estimated structural model.

The structural model was evaluated by examining the path coefficients, coefficients of determination (R^2^), effect size (f^2^), and predictive relevance (Q^2^). The statistical significance of the hypothesized relationships was assessed using a nonparametric bootstrapping procedure with 5,000 resamples, which is recommended for robust inferences in PLS-SEM (Alamer, 2025; Hair et al., 2019) (See Figure 4).

The model’s predictive power was evaluated using the R^2^ values for endogenous constructs, with adjusted R^2^ values reported when multiple predictors were present. Effect sizes (f^2^) were calculated to determine the relative contribution of each exogenous construct (Cohen, 1988). In addition, Stone-Geisser’s Q^2^ values were obtained through a blindfolding procedure to assess the model’s predictive relevance, with values greater than zero indicating a satisfactory predictive capability.

Overall, this analytical approach enabled a rigorous evaluation of both the measurement properties and the hypothesized structural relationships among perceived clarity, trust, engagement, ethical concerns, perceived usefulness, and future readiness for AI-GF in higher-education EFL contexts.

## 5 Results

### 5.1 Pilot testing

Before data collection, a pilot study was conducted with 30 undergraduate EFL students to assess the questionnaire’s reliability. Internal consistency was assessed using Cronbach’s alpha. The 15-item Likert-scale instrument demonstrated excellent reliability (*α* = 0.943), exceeding the commonly accepted threshold of 0.70 (Taber, 2018). This high coefficient indicated strong internal consistency among the items and suggests that the questionnaire reliably measured learners’ perceptions of AI-GF. Based on the satisfactory reliability results from the pilot test, the instrument was deemed suitable for the main study, and full-scale data collection proceeded. The results are reported in relation to the hypothesized structural relationships articulated in the research questions and proposed conceptual framework.

### 5.2 Measurement model evaluation

Before testing the hypothesized relationships, the measurement model was evaluated to establish its construct reliability and validity. Overall, the results indicated that the measurement model demonstrated satisfactory psychometric properties.

Convergent validity was assessed by examining the indicator loadings and average variance extracted (AVE). As Table 2 shows, all standardized factor loadings were statistically significant and exceeded the recommended threshold of 0.50. In addition, the AVE values for all latent constructs surpassed the minimum criterion of 0.50, ranging from 0.521 (Perceived Clarity) to 0.778 (Engagement). These results confirmed that each construct explained an adequate proportion of the variance in its indicators, thereby supporting convergent validity.

**Table 2:** Assessment of convergent validity and reliability of the measurement model.

Latent constructs	Indicators	Factor loading	AVE	CR	Cronbach’s Alpha
Perceived clarity (CL)	CL1	0.267*	0.521	0.760	0.757
	CL2	0.617			
	CL3	0.628			
	CL4	0.888			
Trust in AI feedback (TR)	TR1	0.886	0.738	0.891	0.879
	TR2	0.916			
	TR3	0.880			
	TR4	0.743			
Perceived usefulness (PU)	PU1	0.886	0.767	0.902	0.899
	PU2	0.854			
	PU3	0.885			
	PU4	0.876			
Engagement with AI feedback (ENG)	ENG1	0.889	0.778	0.908	0.904
	ENG2	0.904			
	ENG3	0.914			
	ENG4	0.818			
Ethical concern (EC)	EC1	0.895	0.722	0.878	0.870
	EC2	0.891			
	EC3	0.836			
	EC4	0.770			
Future readiness training (FR)	FR1	0.858	0.675	0.852	0.841
	FR2	0.838			
	FR3	0.810			
	FR4	0.846			

AVE, average variance extracted; CR, composite reliability. ^*^Indicator CL1 was removed due to low factor loading (<0.40).

Discriminant validity was evaluated using both the heterotrait-monotrait ratio (HTMT) and the Fornell-Larcker criterion. As Table 3 shows, all HTMT values are below the conservative threshold of 0.85, indicating satisfactory discriminant validity. Furthermore, the square root of each construct’s AVE exceeds its correlations with other constructs, providing additional evidence that the latent variables are empirically distinct.

**Table 3:** Assessment of discriminant validity.

Latent constructs	CL	TR	PU	ENG	EC
Heterotrait-monotrait ratio (HTMT) criterion					
CL	—				
TR	0.738	—			
PU	0.716	0.763	—		
ENG	0.711	0.818	0.724	—	
EC	0.738	0.832	0.758	0.796	—
FR	0.764	0.759	0.786	0.829	0.813
Fornell-Larcker criterion					
CL	**0.722**				
TR	0.621	**0.859**			
PU	0.667	0.754	**0.876**		
ENG	0.649	0.779	0.684	**0.882**	
EC	−0.662	−0.746	−0.783	−0.803	**0.850**
FR	0.533	0.673	0.692	0.779	−0.767

CL, perceived clarity; TR, trust in AI feedback; PU, perceived usefulness; ENG = engagement with AI feedback; EC, ethical concern. HTMT values below 0.90 indicate adequate discriminant validity.

Internal consistency reliability was examined using Cronbach’s alpha and composite reliability (CR). As presented in Table 2, the Cronbach’s alpha values ranged from 0.757 to 0.904, while the CR values ranged from 0.760 to 0.908. All values exceeded the recommended minimum of 0.70, indicating strong internal consistency across the constructs (Hair et al., 2019). Collectively, these results confirm the measurement model’s reliability and validity, supporting the subsequent structural model evaluation.

### 5.3 Structural model evaluation

The structural model was assessed after confirming model adequacy. Multicollinearity was evaluated using variance inflation factor (VIF) values and inter-construct correlations. As Table 4 shows, all the VIF values were below the conservative threshold of 5.0, indicating that multicollinearity was not a concern. In addition, none of the inter-construct correlations exceeded 0.85, further supporting the absence of collinearity.

**Table 4:** Structural model results showing path coefficients, effect sizes (f^2^), and collinearity statistics (VIF).

Path	β	SD	t-value	*p*-value	*f* ^2^	VIF	Result
CL ➔ PU	0.177	0.049	3.577	0.000	0.080	1.729	H_1_ supported
ENG ➔ PU	0.756	0.042	17.973	0.000	1.471	1.738	H_2_ supported
TR ➔ ENG	0.779	0.036	21.585	0.000	1.548	1.000	H_3_ supported
EC ➔ TR	- 0.746	0.039	19.081	0.000	1.257	1.000	H_4_ supported
PU ➔ FR	0.220	0.086	2.551	0.011	0.026	3.122	H_5_ supported
ENG ➔ FR	0.543	0.084	6.489	0.006	0.159	3.124	H_6_ supported

β = standardized path coefficient; SD = standard deviation; f^2^ = effect size; VIF = variance inflation factor. Significant paths are reported at *p* < 0.05.

### 5.4 Hypothesis testing and structural relationships

Hypothesis testing was conducted using a bootstrapping procedure with 5,000 re-samples. Table 4 presents the standardized path coefficients, t-values, *p*-values, and effect sizes (f^2^). Perceived clarity (CL) exerted a positive and statistically significant effect on perceived usefulness (PU) (*β* = 0.177, t = 3.577, *p* < 0.001, f^2^ = 0.080), providing support for H1. Although statistically significant, the effect size was small, indicating a modest contribution to PU. In contrast, engagement with AI feedback (ENG) showed a strong positive effect on PU (*β* = 0.756, t = 17.973, *p* < 0.001, f^2^ = 1.471), supporting H2 and indicating a substantial explanatory contribution.

Trust in AI-GF (TR) demonstrated a strong positive influence on engagement (ENG) (*β* = 0.779, t = 21.585, *p* < 0.001, f^2^ = 1.548), thereby supporting H3. This result underscores trust as a critical determinant of learner engagement with AI-GF. Ethical concerns (EC) exhibited a significant negative effect on trust (*β* = −0.746, t = 19.081, *p* < 0.001, f^2^ = 1.257), supporting H4 and highlighting the detrimental role of ethical apprehensions in shaping trust perceptions.

Regarding future readiness and training (FR), perceived usefulness (PU) exerted a positive but relatively weak effect (*β* = 0.220, t = 2.551, *p* = 0.011, f^2^ = 0.026), supporting H5. Engagement (ENG), however, had a stronger and statistically significant effect on FR (*β* = 0.543, t = 6.489, *p* < 0.01, f^2^ = 0.159), strongly supporting H6 and indicating that active AI feedback engagement is a key predictor of learners’ readiness for future AI-supported learning.

### 5.5 Predictive power and predictive relevance

The structural model’s explanatory and predictive performances were evaluated using the coefficients of determination (R^2^ and adjusted R^2^) and Stone-Geisser Q^2^ values (Table 5). Ethical concerns explained 55.5% of the variance in trust (R^2^ = 0.555). Perceived clarity and engagement jointly accounted for 77.4% of the variance in perceived usefulness (adjusted R^2^ = 0.774), indicating a substantial explanatory power. Trust explained 60.6% of the variance in engagement (R^2^ = 0.606), while perceived usefulness and engagement together explained 54.7% of the variance in future readiness (adjusted R^2^ = 0.547).

**Table 5:** Assessment of predictive power and predictive relevance.

Dependent variable	R-squared	Adjusted R-squared	*Q^2^*
Trust in AI feedback (TR)	**0.557**	**0.555**	**0.407**
Perceived usefulness (PU)	0.776	0.774	0.584
Engagement with AI feedback (ENG)	0.608	0.606	0.467
Future readiness training (FR)	0.551	0.547	0.353

R-squared = Variance explained by model; Adjusted R-squared = R-squared adjusted for predictors; Q^2^: obtained from the blindfolding procedure based on the cross-validated redundancy approach.

All endogenous constructs exhibited meaningful predictive relevance with Q^2^ values exceeding zero. Perceived usefulness had the highest predictive relevance (Q^2^ = 0.584), followed by engagement (Q^2^ = 0.467), trust (Q^2^ = 0.407), and future readiness (Q^2^ = 0.353). These results indicate that the model has strong in-sample explanatory power and adequate out-of-sample predictive capability.

## 6 Discussion

This study examined how EFL learners perceive and engage with AI-GF through a learner-centered framework that considers trust, ethical concerns, engagement, perceived clarity, perceived usefulness, and future readiness. Rather than focusing solely on technology acceptance or behavioral intention, the study explored how learners’ perceptions and interactions with AI feedback are associated with their engagement and evaluations of its learning value. The findings offer insights into the cognitive, affective, and behavioral dimensions of learner responses to AI-mediated feedback in higher-education EFL contexts and suggest several theoretical and pedagogical considerations for the integration of AI tools in language learning.

### 6.1 Perceived clarity of AI-generated feedback

The findings indicate that learners generally perceived AI-GF as clear and comprehensible, with perceived clarity showing a positive association with perceived usefulness (*β* = 0.177, f^2^ = 0.080). This pattern is broadly consistent with the Technology Acceptance Model (TAM), which suggests that ease of understanding may contribute to perceptions of usefulness (Davis, 1989). Similar observations have been reported in previous studies, where clearer feedback was associated with improved learner interpretation and application of feedback in language learning contexts (Aljasser, 2025).

However, the relatively modest effect size observed in this study suggests that clarity alone may not fully account for learners’ perceptions of educational value. Instead, clarity may function as an enabling condition that supports learners’ ability to interpret feedback, while other factors, such as trust and engagement, appear to be more strongly associated with how learners evaluate its usefulness.

### 6.2 Trust in AI-generated feedback

Trust was strongly associated with engagement with AI-GF (*β* = 0.779, f^2^ = 1.548), accounting for a substantial proportion of variance in learner engagement. Learners who perceived AI feedback as credible, pedagogically coherent, and relevant reported higher levels of cognitive and behavioral interaction with the feedback.

This observation aligns with prior research highlighting trust as an important factor in learners’ willingness to interact with AI systems in educational settings (Qin et al., 2020). While traditional technology acceptance models often position trust primarily as a predictor of usage intention, the present findings suggest that trust may also be closely related to the extent to which learners actively engage with AI-GF during the learning process.

These results indicate that trust may play an important role in shaping learners’ perceptions of AI-supported feedback environments, particularly when learners evaluate the credibility and pedagogical relevance of automated suggestions.

### 6.3 Ethical concerns as barriers to trust

Ethical concerns, including perceptions related to bias, fairness, and potential overreliance on AI systems, showed a substantial negative association with trust (*β* = −0.746, f^2^ = 1.257). This pattern suggests that learners’ ethical perceptions may be closely related to how they evaluate the credibility and reliability of AI-GF.

These findings are consistent with emerging research in AI-supported education, which emphasizes that trust in automated systems is influenced not only by technical accuracy but also by perceptions of transparency, fairness, and responsible AI use (Aljasser, 2025; Corbin et al., 2025a). Similar concerns have been observed in EFL contexts, where learners sometimes express uncertainty regarding the fairness and reliability of automated evaluation systems (Alghamdy, 2023; Aljabr and Al-Ahdal, 2024; Alshamy et al., 2025).

From a theoretical perspective, the results suggest that ethical perceptions may represent an important dimension within AI adoption frameworks in educational settings. Rather than functioning solely as contextual concerns, ethical considerations appear closely associated with learners’ trust in AI-mediated feedback.

It is also important to interpret these findings in light of the study’s demographic composition. The sample was predominantly female (93.7%), reflecting the institutional context in which the study was conducted. Previous research suggests that demographic characteristics, including gender, may shape attitudes toward emerging technologies and perceptions of ethical risks such as fairness, transparency, and responsible AI use. Consequently, the strong association observed between ethical concerns and trust may partly reflect the perspectives of this demographic group. Future studies should therefore examine whether similar patterns emerge in more gender-balanced samples and across diverse educational contexts.

In sum, these findings highlight the importance of addressing ethical transparency and responsible AI practices in educational environments, as ethical perceptions appear closely linked to learners’ trust in AI-GF.

### 6.4 Engagement as the primary pathway to perceived usefulness

Engagement with AI-GF showed a strong positive association with perceived usefulness (*β* = 0.756, f^2^ = 1.471). Learners who reported higher levels of interaction with AI feedback, such as reflecting on suggestions, revising their writing, or integrating feedback into subsequent work, also tended to perceive the feedback as more valuable for their learning.

This observation aligns with constructivist perspectives on learning, which emphasize that educational technologies are most effective when learners actively engage with learning materials and feedback. Similar patterns have been reported in recent research on AI-supported learning environments, where engagement has been linked to more positive evaluations of AI feedback and learning support (Huang and Mizumoto, 2025; Jin et al., 2025; Zhang et al., 2024).

Rather than suggesting a direct causal pathway, these results indicate that learners’ perceptions of usefulness appear closely associated with the degree to which they interact with and reflect upon AI-GF.

### 6.5 Clarity as an enabling but insufficient condition

Although perceived clarity showed a significant relationship with perceived usefulness, its relatively smaller effect size suggests that clarity may function primarily as a supporting factor rather than a primary driver of perceived educational value. Clear feedback may help learners interpret automated suggestions more easily, but clarity alone may not guarantee that learners perceive the feedback as beneficial.

This interpretation aligns with prior research suggesting that linguistic clarity in feedback is important but may need to be accompanied by meaningful engagement and contextual understanding for feedback to support learning effectively (Mirzaei and Khayer, 2025; Zhang et al., 2024).

### 6.6 Predicting future readiness

Both engagement and perceived usefulness were positively associated with learners’ future readiness, with engagement exhibiting a stronger effect (*β* = 0.543, f^2^ = 0.159) than perceived usefulness (*β* = 0.220, f^2^ = 0.026), indicating meaningful predictive power in the structural model. These findings suggest that learners who actively engage with AI-GF are more likely to feel prepared to adopt similar technologies in future academic or professional contexts.

Within the SEM framework, engagement with AI-GF was also linked to broader latent learning constructs, including reflective learning, self-regulation, and digital tool proficiency (Achuthan, 2025; BinJwair and Alamer, 2025; Jwair, 2025; Praveen et al., 2025; Yang and Xia, 2023). Students demonstrating higher engagement scores exhibited greater metacognitive monitoring and autonomous learning strategies, highlighting the role of AI-GF in enhancing self-regulated learning and reflective practices.

Given the cross-sectional design, the observed associations should not be interpreted as causal pathways. Future research employing longitudinal or experimental PLS-SEM designs could elucidate how engagement with AI feedback evolves and its dynamic influence on learners’ broader self-regulated learning behaviors, thereby strengthening the model’s predictive validity in diverse higher education contexts.

### 6.7 Integrated model performance

The structural model demonstrated substantial explanatory and predictive capacity for perceived usefulness (adjusted R^2^ = 0.774, Q^2^ = 0.584) and learner engagement (Q^2^ = 0.467), exceeding commonly accepted thresholds in PLS-SEM research. These results suggest that the proposed framework provides a useful lens for examining how learners perceive and interact with AI-GF in higher-education contexts.

Integrated models that position learner engagement alongside trust and ethical perceptions have also demonstrated strong explanatory potential in similar educational technology studies (Luo and Yusuf, 2025; Naidoo, 2023; Ouyang et al., 2023).

### 6.8 Theoretical contributions

The findings contribute to the growing literature on AI-supported feedback in several ways.

1. The results highlight the importance of learner engagement as a key dimension associated with learners’ perceptions of AI-GF, extending traditional technology acceptance approaches that focus primarily on intention to use.
2. Ethical concerns emerged as strongly associated with trust, suggesting that ethical perceptions may represent an important component of AI adoption frameworks in educational contexts.
3. The findings provide further insight into how multiple learner perceptions, including trust, clarity, engagement, and perceived usefulness, are interrelated within AI-mediated feedback environments.

### 6.9 Pedagogical implications

The results also suggest several implications for educational practice.

1. Institutions introducing AI-GF systems should consider strategies that support the development of trust, including clear explanations of how AI tools generate feedback and how such feedback should be interpreted.
2. Ethical transparency and AI literacy appear important for addressing learners’ concerns about bias, fairness, and responsible AI use. Guiding the appropriate use of AI tools may help learners critically evaluate automated feedback rather than relying on it uncritically.
3. Instructors may benefit from encouraging guided engagement with AI feedback, such as reflective revision activities or discussions about how to interpret automated suggestions.

Overall, the findings suggest that the educational value of AI-GF may depend not only on the technological capabilities of the tools themselves but also on how learners perceive, interpret, and engage with the feedback they receive.

### 6.10 Limitations and future research directions

Despite its theoretical and methodological contributions, this study has several limitations. For example, the reliance on self-reported questionnaire data may introduce biases, including social desirability and subjective interpretation, such that participants’ responses may reflect perceptions of AI-GF rather than actual learning behaviors. Furthermore, the cross-sectional design, while suitable for examining structural relationships using PLS-SEM, limits the ability to infer causal relationships among trust in AI feedback, ethical perceptions, engagement, and perceived learning outcomes. Additionally, the study focused exclusively on EFL learners in Saudi higher education, which may restrict the generalizability of the findings to other educational systems, cultural contexts, or institutions with different technological infrastructures or pedagogical practices. Additionally, the predominantly female sample (93.7%) reflects institutional demographics but may limit the applicability of results to more gender-balanced populations. Finally, only a portion of participants had direct experience with AI-GF, which may influence responses regarding engagement and perceptions.

These limitations point to several directions for future research. Longitudinal or experimental designs could explore how learners’ trust, engagement, and perceptions of AI-GF evolve, providing stronger evidence of causal relationships. Methodological rigor could be enhanced through data triangulation, combining self-reported measures with complementary sources such as learning analytics, system interaction logs, classroom observations, or qualitative interviews, offering a more nuanced understanding of engagement with AI feedback. Replicating and extending the proposed model across diverse educational contexts, institutions, and cultural settings would strengthen external validity. Furthermore, future studies could adopt a multidimensional approach to ethical concerns, distinguishing among dimensions such as algorithmic bias, transparency, privacy protection, and responsible AI use, to examine their specific influence on learners’ trust in and engagement with AI-GF. Finally, future research may investigate the potential moderating effects of prior experience with AI-GF on the relationships among latent constructs in SEM models.

## 7 Conclusion

This study examined EFL learners’ engagement with AI-GF within a learner-centered framework that highlights trust, ethical issues, perceived clarity, engagement, perceived usefulness, and future readiness. The results showed that trust was the primary driver of engagement, with learners engaging more cognitively and behaviorally when they perceived AI feedback as credible, accurate, and pedagogically relevant. Ethical concerns had a strong negative impact on trust, underscoring the importance of ethics in AI adoption. Furthermore, engagement served as the primary pathway through which AI-GF affected perceived usefulness and future readiness, whereas clarity played a secondary, supportive role. Ultimately, both engagement and perceived usefulness contribute to learners’ future readiness, with engagement being a stronger predictor, emphasizing the need for ongoing active interaction with AI feedback.

Theoretically, this article extends the TAM and UTAUT frameworks by positioning engagement as the key outcome and emphasizing ethics and trust as core determinants of AI adoption in education. Pedagogically, the findings suggest that institutions should focus on building trust, implementing ethical safeguards, and providing structured guidance to maximize the educational benefits of AI-GF. Active reflective engagement with AI feedback is essential to convert clarity and technical quality into meaningful learning outcomes.

Despite these insights, this study had several limitations. For instance, the sample was drawn from a single EFL context, which may limit the applicability of the findings to other educational settings and proficiency levels. In addition, this study relied on self-reported data on engagement and perceptions, which can be affected by social desirability or response bias. Moreover, focusing on AI-GF in written tasks may not capture learners’ engagement with other AI-assisted formats, such as oral feedback or interactive tutoring systems.

These limitations open up multiple avenues for future research. Future investigations could explore cross-cultural or multilingual contexts to determine how trust, ethics, and engagement differ among diverse learner groups. Using experimental or mixed-method approaches could provide more objective measures of learning outcomes and behavioral engagement, supplementing self-report data. Future studies could also examine the long-term effects of AI-GF on skill development, motivation, and autonomous learning, as well as how various AI feedback types influence engagement and perceived usefulness.

In summary, this study demonstrates that the success of AI-GF in EFL learning depends not only on technology but also on trust, ethical transparency, and active learner participation. Engagement is the central mechanism that transforms AI feedback into perceived usefulness and future learning readiness, offering valuable insights into the thoughtful and effective integration of AI into higher education.

## Data Availability

The datasets presented in this study are available in online repositories. The names of the repository/repositories and accession number(s) can be found in the article/Supplementary material.
