# Supplementary material for: Trust, ethical, and learning impact of artificial intelligence feedback in English education
Source: Front Psychol. 2026 Apr 16;17:1809661. doi: 10.3389/fpsyg.2026.1809661 (PMC13128619; doi:10.3389/fpsyg.2026.1809661)
Supplement: Supplementary file 1 [file Supplementary_file_1.docx]

1. **Appendix 1**

**Part One: Demographic Information:**

Gender: Male - Female

Age: under18 / 18 +

Year of study: 1st year / 2nd year / 3rd year / 4th year

Academic Program: Diploma / Bachelor's / Master's

Received AI-GF on academic work: Yes- No

**Part Two: Measurement Constructs and Survey Items**

The questionnaire included multiple constructs measuring learners’ perceptions of AI-GF. Each construct was operationalized using four items adapted from previously validated instruments in educational technology and AI-in-education research. Minor wording modifications were made to ensure contextual relevance to AI-GF in EFL higher-education settings.

**Perceived Clarity**

Perceived clarity refers to learners’ perceptions that AI-GF is clear, understandable, and easy to interpret. The items measuring perceived clarity were adapted from Roe et al. (2024) and Zhang et al. (2025). The construct was measured using four items:

- CL1: AI-GF is clear and easy to understand.
- CL2: The instructions and suggestions provided by AI feedback are straightforward.
- CL3: I can easily interpret AI-generated comments on my work.
- CL4: The language used in AI feedback is easy to comprehend.

**Trust in AI Feedback**

Trust in AI feedback reflects learners’ confidence in the reliability, credibility, and pedagogical value of feedback generated by AI systems. The items were adapted from Zhang et al. (2025) and Henderson et al. (2025). The construct was measured using the following four items:

- TR1: I trust the accuracy of AI-GF.
- TR2: AI feedback provides reliable suggestions for improving my work.
- TR3: I perceive AI feedback as objective and unbiased.
- TR4: I feel confident using AI feedback to revise my assignments.

**Perceived Usefulness**

Perceived usefulness captures learners’ perceptions that AI-GF supports their learning performance and contributes to language improvement. The items were adapted from Roe et al. (2024) and Alshamy et al. (2025). The construct was measured through four items:

- PU1: AI feedback helps me improve the quality of my work.
- PU2: AI feedback motivates me to revise my work thoughtfully.
- PU3: Using AI feedback enhances my academic performance.
- PU4: AI feedback saves time in the revision process.

**Engagement with AI Feedback**

Engagement refers to learners’ cognitive and behavioural interaction with AI-GF, including reflection, revision, and the integration of feedback into learning tasks. The items were adapted from Sahari et al. (2025) and Zhang et al. (2025). The construct included the following items:

- ENG1: I critically reflect on AI feedback rather than accepting it at face value.
- ENG2: I combine AI feedback and human feedback when revising assignments.
- ENG3: I actively seek AI feedback to improve my work.
- ENG4: I use AI feedback to set goals for future tasks.

**Ethical Concerns**

Ethical concerns capture learners’ perceptions of fairness, transparency, bias, and responsible use of AI in automated feedback systems. The items were adapted from Corbin et al. (2025) and reports from Axios and The Guardian (2024). The construct was measured through four items:

- EC1: I am concerned that AI feedback may contain inaccuracies or promote overconfidence.
- EC2: AI feedback might undermine my critical thinking or learning depth.
- EC3: I have privacy or ethical concerns when using AI-GF.
- EC4: I worry about the misuse of AI in academic tasks.

**Future Readiness and Training**

Future readiness and training refer to learners’ perceived preparedness to use AI technologies effectively in future academic or professional contexts. The items were adapted from Aljasser (2025) and Zhang et al. (2025). The construct was measured using four items:

- FR1: I would benefit from training on how to interpret AI-GF.
- FR2: My institution should integrate AI literacy and feedback training into the curriculum.
- FR3: I feel prepared to use AI feedback effectively in future tasks.
- FR4: AI training would enhance my confidence in applying feedback to learning.
